# RETRACTION: Changes of Fibrinolytic System in Thrombolytic Resuscitation of Pulmonary Thromboembolism‐Induced Cardiac Arrest Model

**DOI:** 10.1111/iwj.70549

**Published:** 2025-04-18

## Abstract

**RETRACTION:** TongN. and LiC. , “Changes of Fibrinolytic System in Thrombolytic Resuscitation of Pulmonary Thromboembolism‐Induced Cardiac Arrest Model,” International Wound Journal 18, no. 6 (2021): 874–880, 10.1111/iwj.13589.33942504 PMC8613376

The above article, published online on 04 May 2021, in Wiley Online Library (http://onlinelibrary.wiley.com/), has been retracted by agreement between the journal Editor in Chief, Professor Keith Harding; and John Wiley & Sons Ltd. Following an investigation by the publisher, all parties have concluded that this article was accepted solely on the basis of a compromised peer review process. In addition, further investigation by the publisher found that the article's topic was not within the scope of the journal. In view of the clear evidence of compromised peer review, the parties agreed that the paper must be retracted. The authors did not respond to our notice regarding the retraction.

**RETRACTION:** N. Tong and C. Li , “Changes of Fibrinolytic System in Thrombolytic Resuscitation of Pulmonary Thromboembolism‐Induced Cardiac Arrest Model,” International Wound Journal 18, no. 6 (2021): 874–880, 10.1111/iwj.13589.33942504 PMC8613376

The above article, published online on 04 May 2021, in Wiley Online Library (http://onlinelibrary.wiley.com/), has been retracted by agreement between the journal Editor in Chief, Professor Keith Harding; and John Wiley & Sons Ltd. Following an investigation by the publisher, all parties have concluded that this article was accepted solely on the basis of a compromised peer review process. In addition, further investigation by the publisher found that the article's topic was not within the scope of the journal. In view of the clear evidence of compromised peer review, the parties agreed that the paper must be retracted. The authors did not respond to our notice regarding the retraction.

